# Consideration of vaping products as an alternative to adult smoking: a narrative review

**DOI:** 10.1186/s13011-023-00571-w

**Published:** 2023-11-16

**Authors:** Jane A. Foster

**Affiliations:** Department of Psychiatry & Behavioural Neurosciences, St. Joseph’s Healthcare, 50 Charlton Ave. E., Hamilton, ON L8N 4A6 Canada

**Keywords:** Vaping, Non-combustible alternatives, Harm reduction, Electronic cigarette, Smoking

## Abstract

Tobacco harm reduction is a public health approach to reduce the impact of cigarette smoking on individuals. Non-combustible alternatives to cigarettes, such as electronic cigarettes (e-cigarettes), deliver nicotine to the user in the absence of combustion. The absence of combustion in e-cigarettes reduces the level of harmful or potentially harmful chemicals in the aerosol generated. This narrative review examines the published literature that studied the chemistry of e-cigarette aerosols, the related toxicology in cell culture and animal models, as well as clinical studies that investigated short- and long-term changes in biomarkers of smoke exposure after switching to e-cigarettes. In the context of the literature reviewed, the evidence supports the harm reduction potential for adult smokers who switch to e-cigarettes.

## Background

While smoking rates have decreased steadily over the past 20 years, approximately 22% (just less than 1 billion) of people aged 15 + worldwide smoke cigarettes, and smoking-related diseases accounted for 8.7 million deaths worldwide in 2019 [1]. Tobacco harm reduction is a public health approach to reduce the harm associated with smoking cigarettes. This approach provides smokers who do not quit with less harmful nicotine delivery products [2]. Non-combustible alternatives include heated tobacco products, nicotine-containing e-vapor products, and oral nicotine products can serve as options for adult smokers who switch to these alternatives and stop smoking. Heated tobacco products are non-combustible alternatives with electronic heating elements that heat tobacco to generate a tobacco vapor that delivers nicotine to the user (for review, [3]). Oral nicotine products deliver nicotine mainly by absorption through the user’s oral mucosa and include forms with and without tobacco (e.g., Snus and nicotine pouches, respectively) [4]. The non-combustible category of e-vapor products or electronic cigarettes (e-cigarettes) do not contain tobacco; they deliver nicotine to the user when a liquid is heated to form an aerosol (vapor) [4]. This literature review focused on this last type of non-combustible category: e-cigarettes and examined the scientific evidence that investigates whether switching from combustible cigarettes to e-cigarettes has the potential to improve health outcomes for adult smokers.

In the past 5 years, attention to e-cigarettes has increased with approximately 9300 peer-reviewed studies on the topic of vaping products (or e-cigarettes), as well as more than 650 review articles (Web of Science, “e-cigarette,” May 2023). Within the broad scope of research topics in the literature, the papers selected for this review focused on e-cigarettes in the context of key topics related to tobacco harm reduction, including aerosol chemistry studies, toxicological assessments of e-cigarette aerosols with in vitro and in vivo in comparison to cigarette smoke, and clinical investigations that examined the short- and long-term benefits of switching to e-cigarettes for adult smokers (Table 1). By focusing on studies that compare e-cigarettes to cigarettes, the objective of this review is to provide the reader with the current evidence related to the potential health benefits for smokers if they switch to e-cigarettes. Notably, it is important to understand the research evidence on the effects of switching from combustible cigarettes to e-cigarettes at several levels.

**Table 1:** Research studies cited^a^

Year	Design	Main Findings	Reference
2014	Aerosol Chemistry	Reduced levels of HPHCs in aerosol from e-cigarette compared to cigarette smoke	[5]
2016	Aerosol Chemistry	Reduced levels of HPHCs in aerosol from e-cigarette compared to cigarette smoke	[6]
2020	Aerosol Chemistry	Reduced levels of HPHCs in aerosol from e-cigarette compared to cigarette smoke	[7]
2021	Aerosol Chemistry	Reduced levels of HPHCs in aerosol from e-cigarette compared to cigarette smoke	[8, 9]
	In vitro toxicology	No significant or low cytotoxicity of e-cigarette aerosol on human bronchial epithelial BEAS-2B cell line	[9]
2018	Aerosol Chemistry	Low levels of aromatic amines, volatile organic compounds, polycycli aromatic hydrocarbon benzo[a]pyrene in e-liquid and aerosol	[10]
2020	Aerosol Chemistry	Emission levels for most HPHCs were not detectable in 34 commercially available e-cigarettes. Carbonyls including formaldehyde were detected but variable across devices	[11]
2014	In vitro toxicology	No cytotoxicity following exposure to e-cigarette aqueous extracts in human lung epithelial carcinma cells A549. No mutagenic effects in Ames test. No mutagenic effects in micronuclease assay using chinese hamster ovary cells CHO-K1	[12]
2020	In vitro toxicology	No mutagenic effect in Ames test and no genotoxicity in in vitro micronuclease assay following exposure to e-liquids and aerosols. Reduced cytotoxicity of e-cigarette aerosol compared to tobacco smoke	[13]
2016	In vitro toxicology	Reduced cytotoxicity in human lung epithelial cells following exposure to e-cigarette aerosol compared to tobacco smoke	[14]
2017	In vitro toxicology	Compared several e-cigarettes for cytotoxicity and detected both cytotoxic and non-cytotoxic effects	[15]
2016	Cellular and Molecular Changes	No impact of exposure to e-cigarette aerosol on endothelial cell migration compared to cigarette smoke	[16]
2017	Cellular and Molecular Changes	No oxidative stress in human bronchial epithelial cells exposed to e-cigarette aersol extracts	[17]
2016	Cellular and Molecular Changes	No activation of oxidative stress pathways in human coronary artery endothelial cells in response to e-cigarette aerosol compared to cigarette smoke	[18]
2019	Cellular and Molecular Changes	No tissue damage to buccal and small airway cultures, and no impact on cilia beat in small airway cultures following exposure to e-cigarette aerosol. Increased expression of inflammatory genes in buccal cells exposed to e-cigarette aerosols	[19]
2019	Cellular and Molecular Changes	Reduced levels of oxidative stress from exposure to e-cigarette aerosol compared to tobacco smoke on human bronchial epithelial cells. Increased expression of inflammatory mediators	[20]
2019	Cellular and Molecular Changes	No effect of e-cigarette aerosol on airway epithelial morphology or barrier viability. No difference in immune activation between air exposure and e-cigarettee aerosol exposure	[21]
2021	Cellular and Molecular Changes	Biological impact of exposure to e-cigarette reduced in comparison to cigarette smoke including histology, cytotoxicity, cellular function, and gene expression	[22]
2017	Cellular and Molecular Changes	Reduced impact of exposure to e-cigarettes on gene expression compared to cigarette smoke	[23]
2020	Cellular and Molecular Changes	Similar effect of e-cigarette aerosol and cigarette smoke on barrier integrity of airway epithelial cells. No imact on cilia beat frequency in response to exposure to e-cigarette aerosol	[24]
2016	Cellular and Molecular Changes	Differential changes in gene expression in response to exposure to e-cigarette aerosol compared to cigarette smoke. Alterations in glycerophospholipid biosynthesis noted in response to e-cigarette aerosol exposure	[25]
2017	Cellular and Molecular Changes	Reduced cellular and gene expression effects in human bronchial epithelial cells following exposure to e-cigarette aerosol compared to cigarette smoke	[26]
2020	Cellular and Molecular Changes	Increased salivary inflammatory mediator levels in e-cigarette users compared to non-users	[27]
2017	Cellular and Molecular Changes	Reduced bronchial epithelial function in response to exposure to both cigarette smoke and e-cigarette aerosols	[28]
2020	Cellular and Molecular Changes	Impaired in vivo (rats) endothelial function in response to exposure to e-cigarette aerosol	[29]
2016	Cellular and Molecular Changes	Reduced toxic effects of acute e-cigarette aerosol exposure in C57Bl/6 J mice compared to cigarette smoke	[30]
2021	Cellular and Molecular Changes	Increased levels of fibronectin as a measure of tissue injury in B6C3F1 following exposure to e-cigarette aerosol compared to cigarette smoke	[31]
2020	Cellular and Molecular Changes	Exposure to e-cigarette aerosol did not change ceramide profiles or related enzymes in *ApoE-/-* mice	[32]
2020	Cellular and Molecular Changes	Long term (6 month) exposure to e-cigarette aerosol did not compromise bone integrity in *ApoE-/-* mice	[33]
2021	Cellular and Molecular Changes	Reduction effect of exposure to e-cigarette aerosol on lung function and gene expression in *ApoE-/-* mice compared to cigarette smoke	[34]
2020	Cellular and Molecular Changes	Reduced biological response to exposure to e-cigarette aerosol in *ApoE-/-* mice compared to cigarette smoke	[35]
2020	Cellular and Molecular Changes	Similar effect of e-cigarette aerosol and cigarette smoke on oxidative stress and inflammation related to fibrosis	[36]
2020	Cellular and Molecular Changes	Comparable changes in gene expression in Balb/C mice following exposure to e-cigarette aerosol or cigarette smoke	[37]
2021	Cellular and Molecular Changes	Activation of nicotine-related gene expression in brains of mice exposed to e-cigarette aerosol	[38]
2015	Biomarkers of Exposure	Reduced levels of carbon monoxide, nicotine, and acrolein in urine from individuals after switching to e-cigarettes from combustible cigarettes	[39]
2017	Biomarkers of Exposure	Reduced levels of biomarkers of exposure in urine samples from individuals after switching to e-cigarettes from combustible cigarettes	[40]
2018	Biomarkers of Exposure	Nicotine-related in saliva were comparable between e-cigarette users and cigarette users. Urine levels of nicotine were not detectable in e-cigarette users	[41]
2021	Biomarkers of Exposure	No metal detected in the hair samples of e-cigarette users	[42]
2021	Biomarkers of Exposure	Significant reduction in levels of biomarkers of exposure in cigarette users who switched to e-cigarettes. Also reduced in dual users	[43]
2017	Biomarkers of Exposure	Reduced blood and urine levels of toxicants in individuals that switch from combustible cigarettes to e-cigarettes	[44]
2021	Biomarkers of Exposure	Reduced urine levels of NNAL in individuals who switched from combustible cigarettes to e-cigarettes. Also reduced in dual users	[45]

*HPHC* harmful or potentially harmful chemicals, *ApoE* apolipoprotein E-deficient

^a^reviews cited are not included in this table

## E-cigarettes/e-vapor products

### History

**Fig. 1:**
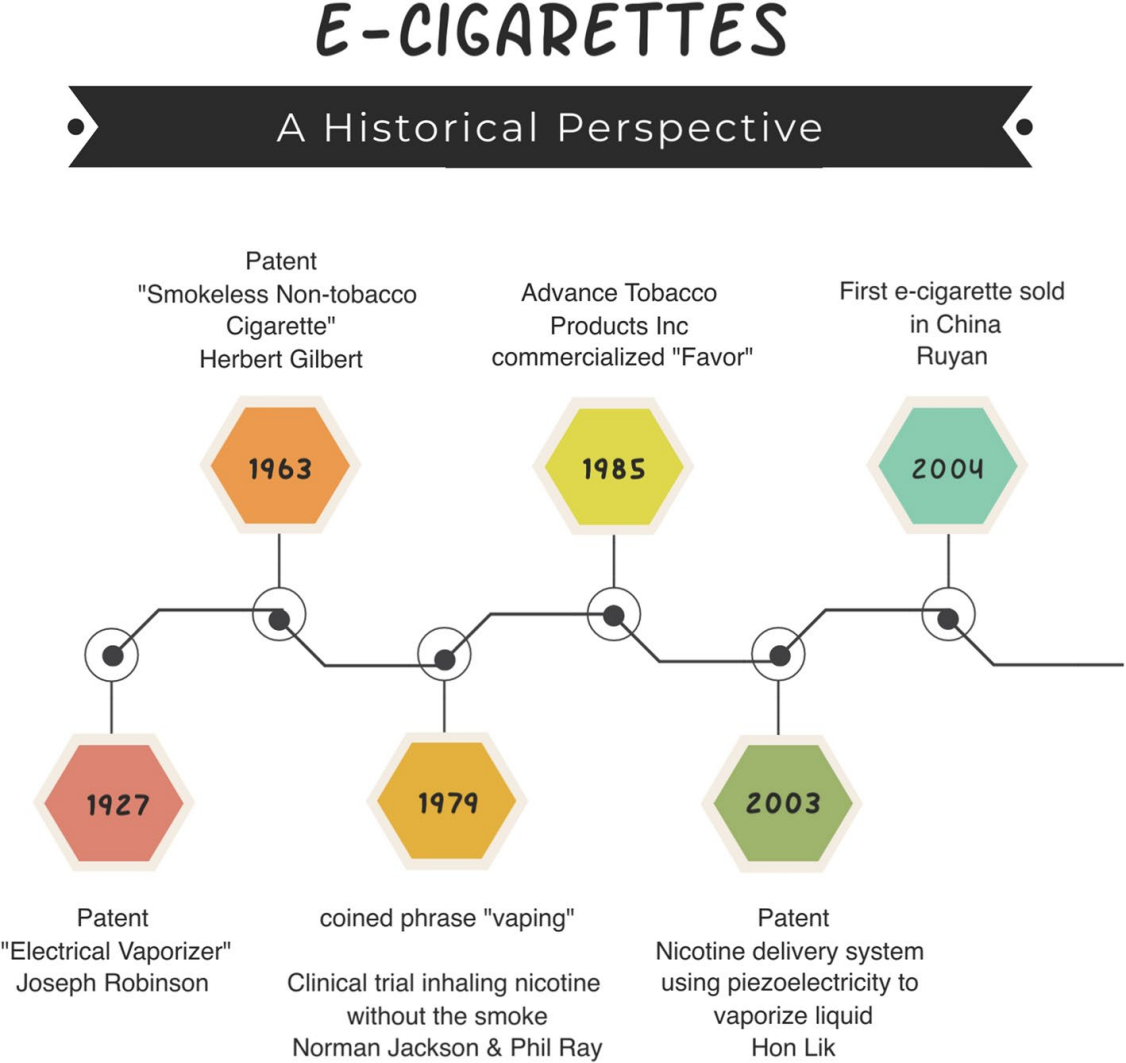
The history of the e-cigarette. Innovation and invention over the past century leading to the development of e-cigarettes, which were first sold in China in 2004. The timeline highlights some landmark events that contributed to the development of modern e-cigarettes (based on [46])

While e-cigarettes are common and most individuals have some knowledge related to e-cigarettes, there are a lot of misconceptions and the history of their development is not well known. The first e-cigarette was sold in China in 2004, but the history of its development goes back to 1927 with the U.S. patent of an “electrical vaporizer” by Joseph Robinson [46]. Other milestones in e-cigarette/e-vapor product development are depicted in Fig. 1 and include the 1963 patent by Herbert Gilbert for a “smokeless non-tobacco cigarette.” In 1979, Phil Ray and Norman Jacobson conducted a clinical trial to test the feasibility of inhaling nicotine without smoke and coined the term “vaping” [46]. In 1985, Advance Tobacco Products Inc. commercialized a version of Ray/Jacobson’s device called “Favor,” but it was banned by the U.S. Food and Drug Administration (FDA) in 1987 [46]. The modern form emerged with the 2003 patent by Hon Lik for a nicotine delivery system that vaporized liquid to deliver nicotine to the user in an aerosol, leading to sales of the first e-cigarette in 2004 in China [46]. The technology of this first device is different from current devices on the market, but all of them use heat to vaporize a liquid and generate a nicotine-containing aerosol.

### Types of e-cigarettes

**Fig. 2:**
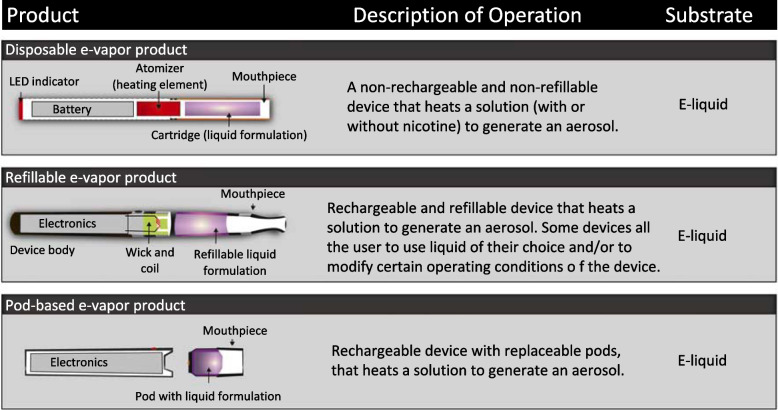
E-cigarette types. Schematics showing the design of disposable, refillable, and pod-based e-cigarettes. (Reproduced with permission from Elsevier [4])

There are three types of e-cigarettes on the market today: disposable self-contained devices, refillable devices, and pod-based devices (Fig. 2). Among these subcategories, there are numerous different devices and a wide range of e-liquid compositions, which makes generalization and comparison challenging. While the main components are similar, it is important to note that some devices are closed, whereas others have open systems that allow the user to add their own e-liquids. The broad range of e-cigarette devices and designs has resulted in regulatory challenges. Following their initial sale in China, e-cigarettes were distributed in Europe and the U.S. in 2006 [47] and are now available worldwide (https://gsthr.org). E-cigarette use has increased in the past decade – there were approximately 58 million e-cigarette users worldwide in 2020, representing 7.1% of the total population, and a sizeable increase from 1.7% estimated in 2012 [48].

## Toxicological assessment of e-cigarette aerosol

Cigarette smoke contains more than 6000 constituents [49], many of which are considered harmful or potentially harmful chemicals (HPHCs). Different regulatory authorities including the World Health Organization, Health Canada, and U.S. FDA have established lists of HPHCs that contribute to smoking-related diseases and should be considered in non-combustible alternative evaluation [8]. It is well-established that combustion of tobacco generates high levels of HPHCs that increase health risks and contribute to smoking-related diseases [50]. A key feature of reduced risk products (RRPs), such as e-cigarettes, is that these devices deliver nicotine to the user in the absence of combustion and hence were designed to reduce exposure to HPHCs compared to cigarette smoke [51]. Investigations reviewed here assessed whether switching to e-cigarettes present less risk than smoking: 1) compare the constituents of the aerosol from e-cigarettes to cigarette smoke, 2) examine the toxicity of aerosols in vitro using tissue culture approaches and in vivo using animal studies, 3) measure HPHC levels in blood and urine from individuals who switch to e-cigarettes and compare them with values in adult smokers, and 4) compare clinical biomarkers of exposure that are known risk factors for smoking-related diseases in individuals who switch to e-cigarettes and those who continue to smoke cigarettes.

Aerosol chemistry studies have shown reduced HPHC levels in some e-cigarette aerosols compared to cigarette smoke [5–7, 9]. Notably, Ruyan, the company that marketed the first e-cigarette in China, reported reduced levels of several known HPHCs in their 2008 safety report [52]. It is important to note the standard lab methodologies for assessing e-cigarette aerosols have continued to be developed in the past decade, and many recent studies use the standards published in the CORESTA E-cigarette Task Force Technical Report [53]. Across several studies, levels of carbonyls and tobacco-specific nitrosamines were reduced by more than 90%. For example, the level of the carcinogen N-nitrosonornicotine (NNN) was detected at 0.05 ng/puff for an e-cigarette compared to 24.9 ng/puff for a reference cigarette [6]. A recent report measured a panel of carbonyl compounds, such as acetaldehyde, acrolein, and formaldehyde, and polycyclic aromatic hydrocarbons classified as carcinogenic or possibly carcinogenic in the aerosols from three e-cigarette products in comparison to cigarette smoke [9]. In this study, all of the carbonyl compounds measured were at very low levels in the e-cigarette aerosols; showing a 99% reduction in total carbonyl content compared to cigarette smoke [9]. Similarly, there was a 92–99% reduction in total polycyclic aromatic hydrocarbons in e-cigarette aerosols compared to cigarette smoke [9]. In a separate study, a comprehensive analysis of combustion-related HPHCs, including aromatic amines, volatile organic compounds, and polycyclic aromatic hydrocarbon benzo[a]pyrene, in e-liquids and their aerosols from commercially available e-cigarettes in the U.S. demonstrated that most of these HPHCs were at very low or below detectable levels in e-liquids or e-cigarette aerosols [10]. Part of the difficulty for the consumer and the non-expert scientist stems from the fact that there are so many different devices on the market, not all research study results apply to every device that is available. The above-noted reports provided evidence of reduced levels of HPHCs in e-cigarette aerosols compared to cigarette smoke, others have raised concerns about the levels of carbonyl compounds such as acetaldehyde, acrolein, and formaldehyde in aerosols generated by heating e-liquids, as well as metals that may be released from the device components [8, 54, 55]. These factors are important to consider and may represent risks associated with e-cigarette use more generally. Moreover, potential risks of excipients in e-liquids and additional potentially harmful chemicals generated from e-liquid heating have garnered attention. E-liquids contain a mixture of propylene glycol, vegetable glycerin, nicotine, flavors, and other constituents. A recent study examined 34 commercially available e-cigarette devices and demonstrated that levels of carbonyls, but particularly formaldehyde, varied across devices, whereas other HPHCs and selected metals were undetectable or very low in the e-cigarette aerosols [11]. The presence of carbonyl compounds, such as formaldehyde, in e-cigarette aerosols results from the heated degradation of e-liquid components (e.g., propylene glycol and glycerin) and depends on device features (e.g., closed vs open systems) and device settings (e.g., heating temperature and voltage) and result in higher HPHC levels in e-cigarette aerosols from some devices [11, 56–59].

Understanding the toxicology of HPHCs in biological systems is an essential component of assessing their risks and benefits. To this end, several investigations have examined the toxicity of e-cigarette aerosols in tissue cultures and animal models. The results of these studies fall into three categories for discussion: 1) no toxicity observed, 2) less toxicity compared to cigarette smoke, and 3) alternative or negative findings.

### In vitro studies

Several studies using tissue culture approaches reported no toxicity following exposure to e-cigarette aerosol, in comparison to the toxic effects observed in the same systems following exposure to cigarette smoke*.* Using an in vitro smoke/aerosol exposure system, exposure to e-cigarette aerosol from two commercial e-cigarettes had no mutagenic (Ames assay) or genotoxic effects (micronucleus assay) [12, 13]. Another group performed scratch wound assays and reported no impact of e-cigarette aerosol on endothelial cell migration, compared to concentration-dependent inhibition following exposure to cigarette smoke [16]. The same group measured intracellular glutathione ratios, oxidant species generation, and activation of nuclear factor erythroid-related factor 2 (Nrf2)-controlled antioxidant response elements and did not detect oxidative stress in human bronchial epithelial cells exposed to e-cigarette aerosol extracts, whereas significant oxidative stress was found in cultures exposed to cigarette smoke [17]. Similarly, activation of the oxidative-stress related transcription factor Nrf2 and cytochrome p450 family member genes was observed in human coronary artery endothelial cells in response to cigarette smoke but not e-cigarette aerosol [18]. Several reports described no tissue damage or molecular changes in buccal and airway epithelial cultures exposed to e-cigarette aerosol compared to alterations observed following exposure to cigarette smoke [9, 19–22]. The above-noted studies showed no detrimental impact of e-cigarette aerosol exposure on the cultures. Additional studies have demonstrated lower toxicity of e-cigarette aerosol compared to cigarette smoke. Significantly reduced (94–99%) cytotoxicity in human bronchial epithelial cells, measured using the neutral red uptake assay, was observed following exposure to e-cigarette aerosols compared to cigarette smoke [13, 14]. Cigarette smoke negatively impacts airway epithelial cell function in vitro [23]. Exposure to e-cigarette aerosol did not impact cilia beat frequency in airway epithelial cells, but it did affect barrier integrity measured with trans-epithelial electric resistance (TEER), albeit to a lower degree than cigarette smoke [22, 23]. A separate study showed a significant impact of e-cigarette aerosol on TEER following 10 days of exposure (compared to 24–48 h in the Haswell study) that was similar to cigarette smoke, suggesting that longer-term exposure to e-cigarette aerosol may have an impact on epithelial barrier function [24]. Differential gene expression analyses in donor-derived differentiated airway epithelial cells exposed to air, 3R4F smoke, or e-cigarette aerosol revealed that smoke induced significant upregulation of 873 RNAs associated with fibrosis, DNA damage signaling, oxidative stress response, and lung cancer [23]. In contrast, 113 differentially expressed RNAs were identified as responsive to the highest concentration of e-cigarette aerosol, but only 3 exceeded a fold change of 2 [23]. Similar findings were observed using RNA-sequencing in differentiated human bronchial epithelial cells and a human bronchial epithelial cell line [20, 25, 26]. Notably, a negative impact of e-cigarette aerosol on inflammatory processes in the cell lines was observed in one of these studies [20]. The potential for e-cigarette use to impact inflammation was recently demonstrated; e-cigarette users (including those who also used marijuana) had higher salivary inflammatory mediator levels compared to non-users [27]. Another group reported that exposure to e-cigarette aerosol was associated with cytotoxicity in human pulmonary fibroblasts, lung epithelial cells, and stem cells, but the authors did not include a cigarette smoke group for comparison [15]. In vitro studies reported impaired endothelial function and reduced epithelial function following exposure to both e-cigarette aerosol and cigarette smoke compared to air, underscoring that e-cigarette aerosol can impact physiological systems [28, 29]. However, in the context of tobacco harm reduction, the collective evidence from numerous assays supports the hypothesis that e-cigarette aerosols are less toxic than cigarette smoke.

### In vivo studies

Animal studies provide important results regarding the biological impact of HPHCs on host systems. Investigators have examined the effects of e-cigarette aerosols on key indicators in animal models known to be affected following exposure to cigarette smoke, including tissue histology, gene expression, cardiovascular function, oxidative stress, and inflammation [30–38, 60]. Mice exposed to e-cigarette aerosols showed a reduced level of lung inflammation and a lower impact on cell proliferation compared to the cigarette smoke-exposed group [30]. In contrast, Sun and colleagues reported a comparable or greater number of histological lung lesions in mice exposed to e-cigarette aerosols versus cigarette smoke; an effect that was suggested to be associated with the acute increase in oxidative stress [31]. Atherosclerosis-prone apolipoprotein E-deficient (*Apoe-/-)* mice are used as an animal model of atherosclerosis and more generally for understanding the pathophysiology of cardiovascular diseases [60]. A recent series of studies in this model system demonstrated reduced cardiovascular effects from exposure to e-cigarette aerosol compared to cigarette smoke, as well as smaller effects on biomarkers of exposure [32–35]. Long-term exposure up to 6 months yielded the expected negative effects of cigarette smoke on lung histology and function, as well as molecular or inflammatory changes. Conversely, no differences in lung function or histopathological changes were observed following exposure to e-cigarette aerosol, and less lung inflammation was observed in *Apoe-/-* mice [34, 35]. In contrast, Ponzoni and colleagues reported similar alterations in gene expression in mice exposed to e-cigarette aerosol or cigarette smoke [37]. Another group reported greater negative cardiac effects in rats exposed to e-cigarette aerosols compared to cigarette smoke [36]. One recent study found a significant impact of long-term exposure to e-cigarette aerosol on nicotine levels and nicotine-related gene expression in the mouse brain compared to air (no comparison to smoke), highlighting the importance of determining the potential risks associated with continued use of nicotine over time [38]. Certainly, longitudinal studies are needed to clarify the acute effects of e-cigarette aerosols.

### Clinical studies

Beyond cell culture and animal studies, it is critical to measure HPHC levels in human biospecimens such as saliva, blood, and urine to provide critical evidence of the potential for reduced risk for adult smokers who completely switch to e-cigarettes. Using a within-subject study design, biomarkers of exposure, including carbon monoxide, nicotine, and acrolein, were measured in urine from smokers before and after switching to e-cigarettes. The results showed reduced levels of all biomarkers at 4 weeks after switching, as well as reduced levels in dual users who did not completely switch to exclusive e-cigarette use [39]. In a similar study, an examination of 7 nicotine metabolites and 17 HPHCs in urine of smokers before and 2 weeks after switching to e-cigarettes revealed that nicotine and some polycyclic aromatic metabolites remained the same after 2 weeks; however, levels of most HPHCs were significantly decreased [40]. In a cross-sectional study that focused on HPHCs in salivary and urine samples, detected salivary levels of *N*^1^-nitrosonornicotine (NNN) in e-cigarette users overlapped with those measured in cigarette smokers, but urine levels of NNN were very low or not detectable in e-cigarette users [41]. As noted above, concern about exposure to metals from the metallic heating device in e-cigarettes warrants attention. A study that detected metals in e-liquid and aerosols from e-cigarettes reported increased levels of copper, chromium, tin, and lead in urine of e-cigarette and dual users compared to non-users; however, no metals were detected in hair samples of e-cigarette users [42].

Clinical studies have also been undertaken to measure biomarkers of exposure that are known risk factors for smoking-related diseases in individuals who switch to e-cigarettes compared to those who continue to smoke cigarettes. Biomarkers of exposure provide quantifiable measures of biological changes in individuals who smoke. Assessing changes in these biomarkers in individuals who switch to RRPs is central to demonstrating the potential for harm reduction for the individual. Blood carboxyhemoglobin—a biomarker for the HPHC carbon monoxide—was reduced by 70–97% as soon as 5 days after switching to e-cigarettes [43, 44]. Notably, 14 of the 23 biomarkers of exposure were significantly reduced in adults who switched to e-cigarettes compared to their baseline levels measured when they were smoking cigarettes [43, 44]. Another study demonstrated reduced urinary levels of 4-(methylnitrosamino)-1-(3-pyridyl)-1-butanol (NNAL) in smokers who switched to e-cigarettes exclusively, as well as in dual users who reduced the number of combustible cigarettes over the 24-week study [45]. The results of clinical studies showing reduced levels of HPHCs and biomarkers of exposure in adult smokers who switch to e-cigarettes provide a base of evidence of the role these products can play in tobacco harm reduction.

 User surveys suggested that the benefits of using of e-cigarettes by smokers included less cigarette consumption, help with smoking cessation, and reduced craving and withdrawal symptoms [61, 62]. A recent study examined the safety profile of e-cigarette use over a 2-year period and demonstrated reduced exposure to HPHCs, and use was not associated with any clinical health concerns including lung function and nicotine withdrawal effects [63]. However, other clinical studies investigating short-term effects on cardiovascular and lung function in e-cigarette users highlighted potential risks associated with e-cigarette use. One study examined vascular function, which is associated with cardiovascular disease, was similar between cigarette smokers and sole e-cigarette users [64]. Another study showed similar increased levels of heart rate variability in cigarette and e-cigarette users, but acute blood pressure increases observed in cigarette smokers were not found in e-cigarette users [65]. In contrast, Barna and colleagues demonstrated that e-cigarette use had no effect on respiratory function measured as persistent alveolitis, which was evident in cigarette smokers [66]. Importantly, individual differences in smoking behavior and other lifestyle factors need to be considered when assessing the risks and benefits of e-cigarette use.

## Conclusions

In summary, there is significant evidence to support the role of e-cigarettes in tobacco harm reduction, but these non-combustible alternatives are not risk free. The long-term risks associated with cigarette smoking are well established, and the best choice for adult smokers is to quit smoking. That said, for individuals who are not able to quit, non-combustible alternatives such as e-cigarettes represent an excellent alternative. While the long-term epidemiological data related to alternatives such as e-cigarettes are not yet available, cancer and non-cancer disease risk estimates for long-term use of non-combustible devices suggest reduced disease risk compared to cigarette smoking [67, 68]. Moving forward, more research is needed to better understand the long-term impact of e-cigarettes on biomarkers of exposure, as well as the effects of long-term e-cigarette use on cardiovascular health and disease outcomes.

## Data Availability

Not applicable.

## Abbreviations

Apoe: Apolipoprotein E
E-cigarette: Electronic cigarette
FDA: Food & Drug Administration
HPHC: Harmful or potentially harmful chemical
NNAL: 4-Methylnitrosamino)-1-(3-pyridyl)-1 butanol
NNN: *N*^1^-Nitrosonornicotine
Nrf2: Nuclear factor erythroid factor 2
RRP: Reduced risk product
TEER: Trans-epithelial electric resistance

## References

[1] WHO. WHO report on the global tobacco epidemic 2021: addressing new and emerging products. World Health Organization. Geneva; 2021.

[2] Hatsukami DK, Carroll DM (2020). Tobacco harm reduction: Past history, current controversies and a proposed approach for the future. Prev Med.

[3] Breheny D, Adamson J, Azzopardi D, Baxter A, Bishop E, Carr T (2017). A novel hybrid tobacco product that delivers a tobacco flavour note with vapour aerosol (Part 2): In vitro biological assessment and comparison with different tobacco-heating products. Food Chem Toxicol.

[4] Smith M, Peitsch MC, Maeder S. Electronic nicotine delivery products. In: Toxicological evaluation of electronic nicotine delivery products [Internet]. London: Academic Press; 2021. 17–22.

[5] Goniewicz ML, Knysak J, Gawron M, Kosmider L, Sobczak A, Kurek J (2014). Levels of selected carcinogens and toxicants in vapour from electronic cigarettes. Tob Control.

[6] Margham J, McAdam K, Forster M, Liu C, Wright C, Mariner D, Proctor C (2016). Chemical composition of aerosol from an e-cigarette: a quantitative comparison with cigarette smoke. Chem Res Toxicol.

[7] Bitzer ZT, Goel R, Trushin N, Muscat J, Richie JP (2020). Free radical production and characterization of heat-not-burn cigarettes in comparison to conventional and electronic cigarettes. Chem Res Toxicol.

[8] Bentley M, Maeder S. Quantification of HPHCs in ENDP Aerosols. In: Toxicological evaluation of electronic nicotine delivery products [Internet]. London: Academic Press; 2021. 41–81.

[9] Dusautoir R, Zarcone G, Verriele M, Garcon G, Fronval I, Beauval N (2021). Comparison of the chemical composition of aerosols from heated tobacco products, electronic cigarettes and tobacco cigarettes and their toxic impacts on the human bronchial epithelial BEAS-2B cells. J Hazard Mater.

[10] Wagner KA, Flora JW, Melvin MS, Avery KC, Ballentine RM, Brown AP, McKinney WJ (2018). An evaluation of electronic cigarette formulations and aerosols for harmful and potentially harmful constituents (HPHCs) typically derived from combustion. Regul Toxicol Pharmacol.

[11] Belushkin M, Tafin Djoko D, Esposito M, Korneliou A, Jeannet C, Lazzerini M, Jaccard G (2020). Selected harmful and potentially harmful constituents levels in commercial e-cigarettes. Chem Res Toxicol.

[12] Misra M, Leverette RD, Cooper BT, Bennett MB, Brown SE (2014). Comparative in vitro toxicity profile of electronic and tobacco cigarettes, smokeless tobacco and nicotine replacement therapy products: e-liquids, extracts and collected aerosols. Int J Environ Res Public Health.

[13] Wieczorek R, Phillips G, Czekala L, Trelles Sticken E, O'Connell G, Simms L (2020). A comparative in vitro toxicity assessment of electronic vaping product e-liquids and aerosols with tobacco cigarette smoke. Toxicol In Vitro.

[14] Azzopardi D, Patel K, Jaunky T, Santopietro S, Camacho OM, McAughey J, Gaca M (2016). Electronic cigarette aerosol induces significantly less cytotoxicity than tobacco smoke. Toxicol Mech Methods.

[15] Behar RZ, Luo W, Lin SC, Wang Y, Valle J, Pankow JF, Talbot P. Distribution, quantification and toxicity of cinnamaldehyde in electronic cigarette refill fluids and aerosols. Tob Control. 2016;25(Suppl 2):ii94-ii102. 10.1136/tobaccocontrol-2016-053224 PMC5503843 27633763

[16] Taylor M, Jaunky T, Hewitt K, Breheny D, Lowe F, Fearon IM, Gaca M (2017). A comparative assessment of e-cigarette aerosols and cigarette smoke on in vitro endothelial cell migration. Toxicol Lett.

[17] Taylor M, Carr T, Oke O, Jaunky T, Breheny D, Lowe F, Gaca M (2016). E-cigarette aerosols induce lower oxidative stress in vitro when compared to tobacco smoke. Toxicol Mech Methods.

[18] Teasdale JE, Newby AC, Timpson NJ, Munafo MR, White SJ (2016). Cigarette smoke but not electronic cigarette aerosol activates a stress response in human coronary artery endothelial cells in culture. Drug Alcohol Depend.

[19] Iskandar AR, Zanetti F, Kondylis A, Martin F, Leroy P, Majeed S (2019). A lower impact of an acute exposure to electronic cigarette aerosols than to cigarette smoke in human organotypic buccal and small airway cultures was demonstrated using systems toxicology assessment. Intern Emerg Med.

[20] Iskandar AR, Zanetti F, Marescotti D, Titz B, Sewer A, Kondylis A (2019). Application of a multi-layer systems toxicology framework for in vitro assessment of the biological effects of Classic Tobacco e-liquid and its corresponding aerosol using an e-cigarette device with MESH technology. Arch Toxicol.

[21] Czekala L, Simms L, Stevenson M, Trelles-Sticken E, Walker P, Walele T (2019). High Content Screening in NHBE cells shows significantly reduced biological activity of flavoured e-liquids, when compared to cigarette smoke condensate. Toxicol In Vitro.

[22] Giralt A, Iskandar AR, Martin F, Moschini E, Serchi T, Kondylis A (2021). Comparison of the biological impact of aerosol of e-vapor device with MESH(R) technology and cigarette smoke on human bronchial and alveolar cultures. Toxicol Lett.

[23] Haswell LE, Baxter A, Banerjee A, Verrastro I, Mushonganono J, Adamson J (2017). Reduced biological effect of e-cigarette aerosol compared to cigarette smoke evaluated in vitro using normalized nicotine dose and RNA-seq-based toxicogenomics. Sci Rep.

[24] Ghosh B, Reyes-Caballero H, Akgun-Olmez SG, Nishida K, Chandrala L, Smirnova L (2020). Effect of sub-chronic exposure to cigarette smoke, electronic cigarette and waterpipe on human lung epithelial barrier function. BMC Pulm Med.

[25] Antherieu S, Garat A, Beauval N, Soyez M, Allorge D, Garcon G, Lo-Guidice JM (2017). Comparison of cellular and transcriptomic effects between electronic cigarette vapor and cigarette smoke in human bronchial epithelial cells. Toxicol In Vitro.

[26] Shen Y, Wolkowicz MJ, Kotova T, Fan L, Timko MP (2016). Transcriptome sequencing reveals e-cigarette vapor and mainstream-smoke from tobacco cigarettes activate different gene expression profiles in human bronchial epithelial cells. Sci Rep.

[27] Ashford K, McCubbin A, Rayens MK, Wiggins A, Dougherty K, Sturgill J, Ickes M (2020). ENDS use among college students: Salivary biomarkers and persistent cough. Addict Behav.

[28] Aufderheide M, Emura M (2017). Phenotypical changes in a differentiating immortalized bronchial epithelial cell line after exposure to mainstream cigarette smoke and e-cigarette vapor. Exp Toxicol Pathol.

[29] Rao DR, Liu J, Springer, ML. JUUL and combustible cigarettes comparably impair endothelial function. Tob Regul Sci. 2020;6(1):30–7. 10.18001/TRS.6.1.4 PMC6953758 31930162

[30] Husari A, Shihadeh A, Talih S, Hashem Y, El Sabban M, Zaatari G (2016). Acute exposure to electronic and combustible cigarette aerosols: effects in an animal model and in human alveolar cells. Nicotine Tob Res.

[31] Sun YW, Chen KM, Atkins H, Aliaga C, Gordon T, Guttenplan JB, El-Bayoumy K (2021). Effects of e-cigarette aerosols with varying levels of nicotine on biomarkers of oxidative stress and inflammation in mice. Chem Res Toxicol.

[32] Lavrynenko O, Titz B, Dijon S, Santos DD, Nury C, Schneider T (2020). Ceramide ratios are affected by cigarette smoke but not heat-not-burn or e-vapor aerosols across four independent mouse studies. Life Sci.

[33] Reumann MK, Schaefer J, Titz B, Aspera-Werz RH, Wong ET, Szostak J (2020). E-vapor aerosols do not compromise bone integrity relative to cigarette smoke after 6-month inhalation in an ApoE(-/-) mouse model. Arch Toxicol.

[34] Szostak J, Wong ET, Titz B, Lee T, Wong SK, Low T (2020). A 6-month systems toxicology inhalation study in ApoE(-/-) mice demonstrates reduced cardiovascular effects of E-vapor aerosols compared with cigarette smoke. Am J Physiol Heart Circ Physiol.

[35] Wong ET, Szostak J, Titz B, Lee T, Wong SK, Lavrynenko O (2021). A 6-month inhalation toxicology study in Apoe(-/-) mice demonstrates substantially lower effects of e-vapor aerosol compared with cigarette smoke in the respiratory tract. Arch Toxicol.

[36] Mayyas F, Aldawod H, Alzoubi KH, Khabour O, Shihadeh A, Eissenberg T (2020). Comparison of the cardiac effects of electronic cigarette aerosol exposure with waterpipe and combustible cigarette smoke exposure in rats. Life Sci.

[37] Ponzoni L, Braida D, Carboni L, Moretti M, Viani P, Clementi F (2020). Persistent cognitive and affective alterations at late withdrawal stages after long-term intermittent exposure to tobacco smoke or electronic cigarette vapour: Behavioural changes and their neurochemical correlates. Pharmacol Res.

[38] Alasmari F, Crotty Alexander LE, Hammad AM, Horton A, Alhaddad H, Schiefer IT (2021). E-cigarette aerosols containing nicotine modulate nicotinic acetylcholine receptors and astroglial glutamate transporters in mesocorticolimbic brain regions of chronically exposed mice. Chem Biol Interact.

[39] McRobbie H, Phillips A, Goniewicz ML, Smith KM, Knight-West O, Przulj D, Hajek P (2015). Effects of switching to electronic cigarettes with and without concurrent smoking on exposure to nicotine, carbon monoxide, and acrolein. Cancer Prev Res (Phila).

[40] Goniewicz ML, Gawron M, Smith DM, Peng M, Jacob P, Benowitz NL (2017). Exposure to nicotine and selected toxicants in cigarette smokers who switched to electronic cigarettes: a longitudinal within-subjects observational study. Nicotine Tob Res.

[41] Bustamante G, Ma B, Yakovlev G, Yershova K, Le C, Jensen J (2018). Presence of the carcinogen n'-nitrosonornicotine in saliva of e-cigarette users. Chem Res Toxicol.

[42] Olmedo P, Rodrigo L, Grau-Perez M, Hilpert M, Navas-Acien A, Tellez-Plaza M (2021). Metal exposure and biomarker levels among e-cigarette users in Spain. Environ Res.

[43] Morris P, McDermott S, Chapman F, Verron T, Cahours X, Stevenson M, et al. Reductions in biomarkers of exposure to selected harmful and potentially harmful constituents following exclusive and partial switching from combustible cigarettes to myblu() electronic nicotine delivery systems (ENDS). Intern Emerg Med. 2021. 10.1007/s11739-021-02813-w PMC8964552 34435305

[44] Round EK, Chen P, Taylor AK, Schmidt E (2019). Biomarkers of tobacco exposure decrease after smokers switch to an e-cigarette or nicotine gum. Nicotine Tob Res.

[45] Cobb CO, Foulds J, Yen MS, Veldheer S, Lopez AA, Yingst JM (2021). Effect of an electronic nicotine delivery system with 0, 8, or 36 mg/mL liquid nicotine versus a cigarette substitute on tobacco-related toxicant exposure: a four-arm, parallel-group, randomised, controlled trial. Lancet Respir Med.

[46] Knowledg-action-change. No fire, no smoke: the global state of tobacco harm reduction. 2018 [Available from: https://gsthr.org/report/full-report (Accessed 30 Dec 2021).

[47] CASAA. Historical timeline of electronic cigarettes: consumer advocates for smoke-free alternatives association; 2021 [Available from: https://casaa.org/education/vaping/historical-timeline-of-electronic-cigarettes/.

[48] Jerzynski T, Stimson GV, Shapiro H, Krol G (2021). Estimation of the global number of e-cigarette users in 2020. Harm Reduct J.

[49] Rodgman A, Perfetti TA (2013). The chemical components of tobacco and tobacco smoke.

[50] Onor IO, Stirling DL, Williams SR, Bediako D, Borghol A, Harris MB, et al. Clinical effects of cigarette smoking: epidemiologic impact and review of pharmacotherapy options. Int J Environ Res Public Health. 2017;14(10):1147. 10.3390/ijerph14101147 PMC5664648 28956852

[51] Peitsch M, Hoeng J (2021). Toxicological evaluation of electronic nicotine delivery products.

[52] Laugesen M. Safety Report on the Ruyan e-cigarette cartridge and inhaled aerosol 2008 [updated October 30, 2008.

[53] CORESTA. Routine analytical machine for E-cigarette aerosol generation and collection - Definitions and standard conditions. 2015.

[54] Eshraghian EA, Al-Delaimy WK (2021). A review of constituents identified in e-cigarette liquids and aerosols. Tob Prev Cessat.

[55] Arnold C (2018). Between the tank and the coil: assessing how metals end up in e-cigarette liquid and vapor. Environ Health Perspect.

[56] Ward AM, Yaman R, Ebbert JO. Electronic nicotine delivery system design and aerosol toxicants: a systematic review. PLoS One. 2020;15(6):e0234189. 10.1371/journal.pone.0234189 PMC7272070 32497139

[57] Son Y, Bhattarai C, Samburova V, Khlystov A. Carbonyls and carbon monoxide emissions from electronic cigarettes affected by device type and use patterns. Int J Environ Res Public Health. 2020;17(8):2767. 10.3390/ijerph17082767 PMC7215697 32316435

[58] Talih S, Salman R, Soule E, El-Hage R, Karam E, Karaoghlanian N, et al. Electrical features, liquid composition and toxicant emissions from 'pod-mod'-like disposable electronic cigarettes. Tob Control. 2022;31:667–70. 10.1136/tobaccocontrol-2020-056362 PMC8586044 33980722

[59] Li Y, Burns AE, Tran LN, Abellar KA, Poindexter M, Li X (2021). Impact of e-liquid composition, coil temperature, and puff topography on the aerosol chemistry of electronic cigarettes. Chem Res Toxicol.

[60] Lo Sasso G, Schlage WK, Boue S, Veljkovic E, Peitsch MC, Hoeng J (2016). The Apoe(-/-) mouse model: a suitable model to study cardiovascular and respiratory diseases in the context of cigarette smoke exposure and harm reduction. J Transl Med.

[61] Etter JF, Bullen C (2011). Electronic cigarette: users profile, utilization, satisfaction and perceived efficacy. Addiction.

[62] Siegel MB, Tanwar KL, Wood KS (2011). Electronic cigarettes as a smoking-cessation: tool results from an online survey. Am J Prev Med.

[63] Walele T, Bush J, Koch A, Savioz R, Martin C, O'Connell G (2018). Evaluation of the safety profile of an electronic vapour product used for two years by smokers in a real-life setting. Regul Toxicol Pharmacol.

[64] Fetterman JL, Keith RJ, Palmisano JN, McGlasson KL, Weisbrod RM, Majid S, et al. Alterations in vascular function associated with the use of combustible and electronic cigarettes. J Am Heart Assoc. 2020;9(9):e014570. 10.1161/JAHA.119.014570 PMC7428567 32345096

[65] Arastoo S, Haptonstall KP, Choroomi Y, Moheimani R, Nguyen K, Tran E (2020). Acute and chronic sympathomimetic effects of e-cigarette and tobacco cigarette smoking: role of nicotine and non-nicotine constituents. Am J Physiol Heart Circ Physiol.

[66] Barna S, Rozsa D, Varga J, Fodor A, Szilasi M, Galuska L, Garai I (2019). First comparative results about the direct effect of traditional cigarette and e-cigarette smoking on lung alveolocapillary membrane using dynamic ventilation scintigraphy. Nucl Med Commun.

[67] Rodrigo G, Jaccard G, Tafin Djoko D, Korneliou A, Esposito M, Belushkin M (2021). Cancer potencies and margin of exposure used for comparative risk assessment of heated tobacco products and electronic cigarettes aerosols with cigarette smoke. Arch Toxicol.

[68] Stephens WE. Comparing the cancer potencies of emissions from vapourised nicotine products including e-cigarettes with those of tobacco smoke. Tob Control. 2018;27:10–7. 10.1136/tobaccocontrol-2017-053808 28778971

